# Supplementary material for: SARS-CoV-2 infection induces mixed M1/M2 phenotype in circulating monocytes and alterations in both dendritic cell and monocyte subsets
Source: PLoS One. 2020 Dec 31;15(12):e0241097. doi: 10.1371/journal.pone.0241097 (PMC7774986; doi:10.1371/journal.pone.0241097)
Supplement: S1 Table — The numbers represent the means ± standard deviations. (DOCX) [file pone.0241097.s001.docx]

**S1 Table.** Haematological and serum biochemistry parameters in COVID-19 patients

| ***Laboratory**** | ***Values*** |  | ***Normal range*** |
| --- | --- | --- | --- |
| **WBC** x10^6^/mL | 7.4 ± 4.2 | | 3.7 – 10.0 |
| **Granulocytes** x10^6^/mL (%) | 5.4 ± 4.0 (74.4) | | 2.0 – 7.0 (44 – 72) |
| **Lymphocytes** x10^6^/mL (%) | 0.8 ± 0.4 (15.5) | | 0.8 – 4.0 (20 – 46) |
| **Monocytes** x10^6^/mL (%) | 0.5 ± 0.3 (8.7) | | 0.12 – 1.2 (2 – 12) |
| **RBC** x10^12^/L | 4.5 ± 0.7 | | 3.9 – 5.1 |
| **PLT**x10^9^/L | 243.5 ± 112.5 | | 135 - 450 |
| **Hgb** g/L | 132.2 ± 20.4 | | 110 - 157 |
| **Glycemia** mmol/L | 8.7 ± 15.7 | | 3.8 – 6.1 |
| **Urea** mmol/L | 6.2 ± 2.8 | | 3 -8 |
| **Creatinin** μmol/L | 83.8 ± 23.5 | | 49 - 106 |
| **AST** IU/L | 42.7 ± 26.2 | | 0 - 40 |
| **ALT** IU/L | 51.8 ± 45.6 | | 0 - 40 |
| **Albumin** g/l | 33.3 ± 6.4 | | 35 - 52 |
| **LDH** U/L | 676.6 ± 313.9 | | 220 - 450 |
| **CK** U/L | 164.6 ± 143.4 | | <190 |
| **D-dimer** ng/mL | 4.8 ± 17.1 | | <0.5 |
| **CRP** mg/L | 75.6 ± 75.4 | | <5.0 |
| **PCT** ng/mL | 0.2 ± 0.2 | | <0.5 |
| **pO_2_** kPa | 9.2 ± 2.3 | | 8.8 – 13.3 |
| **pCO_2_** kPa | 5.3 ± 2.2 | | 4.7 - 6 |
| **Saturation** % | 92.8 ± 6.0 | | 95 - 97 |
| **pH** | 7.4 ± 0.1 | | 7.4 – 7.4 |
| **K** mmol/L | 4.2 ± 0.6 | | 3.5 – 5.3 |
| **Na** mmol/L | 137.3 ±4.9 | | 137 - 147 |

The numbers represent the means ± standard deviations
