# Supplementary figures and images for: SARS-CoV-2 infection induces mixed M1/M2 phenotype in circulating monocytes and alterations in both dendritic cell and monocyte subsets
Source: PLoS One. 2020 Dec 31;15(12):e0241097. doi: 10.1371/journal.pone.0241097 (PMC7774986; doi:10.1371/journal.pone.0241097)

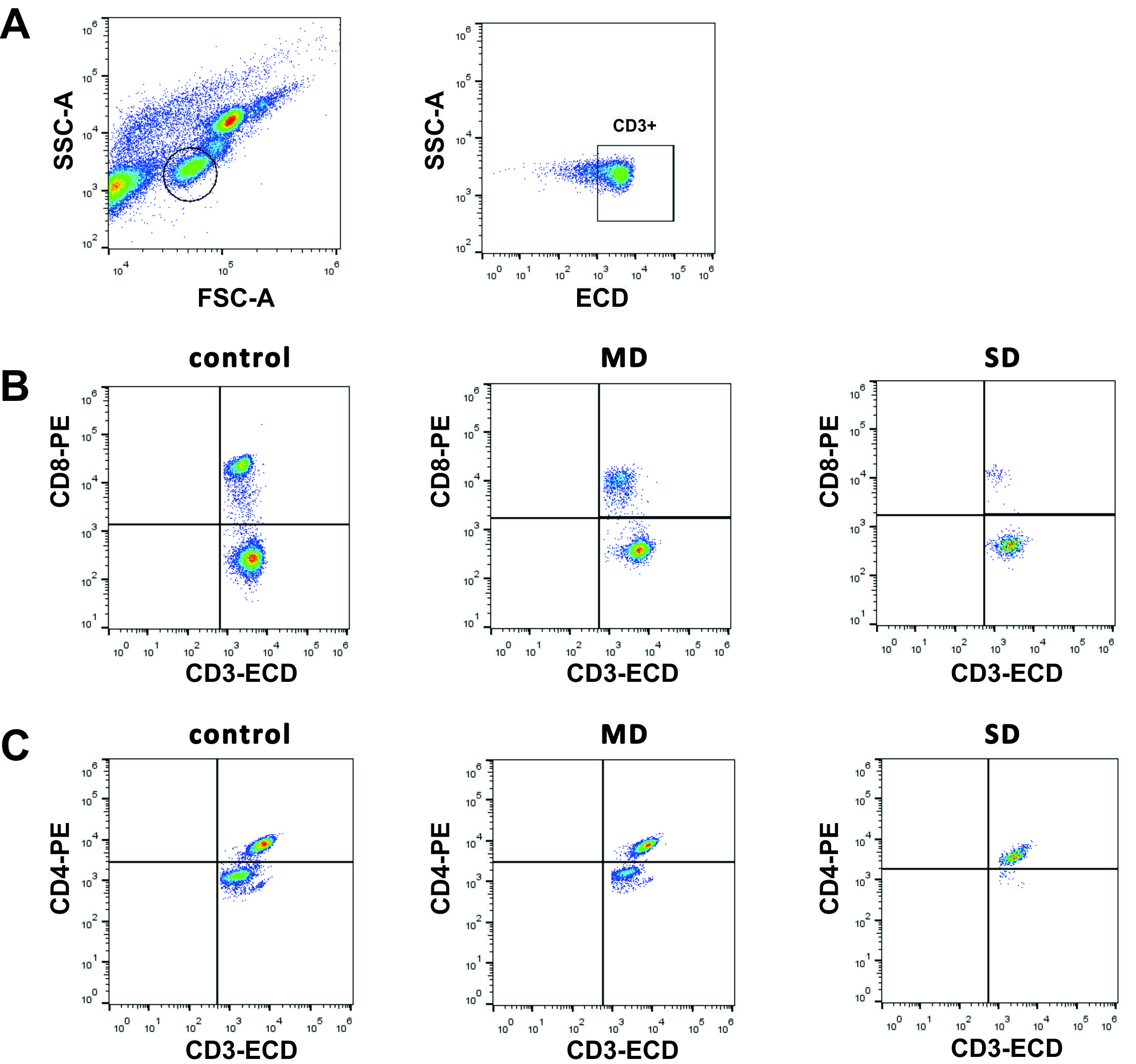

Supplement: S1 Fig — (A) Gating strategy: lymphocytes were selected using FS/SS properties; T lymphocytes were identified based on expression of CD3. (B) CD3 vs. CD8 pseudocolor plots showing CD3+CD8+ cells and (C) CD3 vs. CD4 pseudocolor plots showing CD3+CD4+ cells in T lymphocyte population of controls and patients. (TIF) [file pone.0241097.s002.tif]

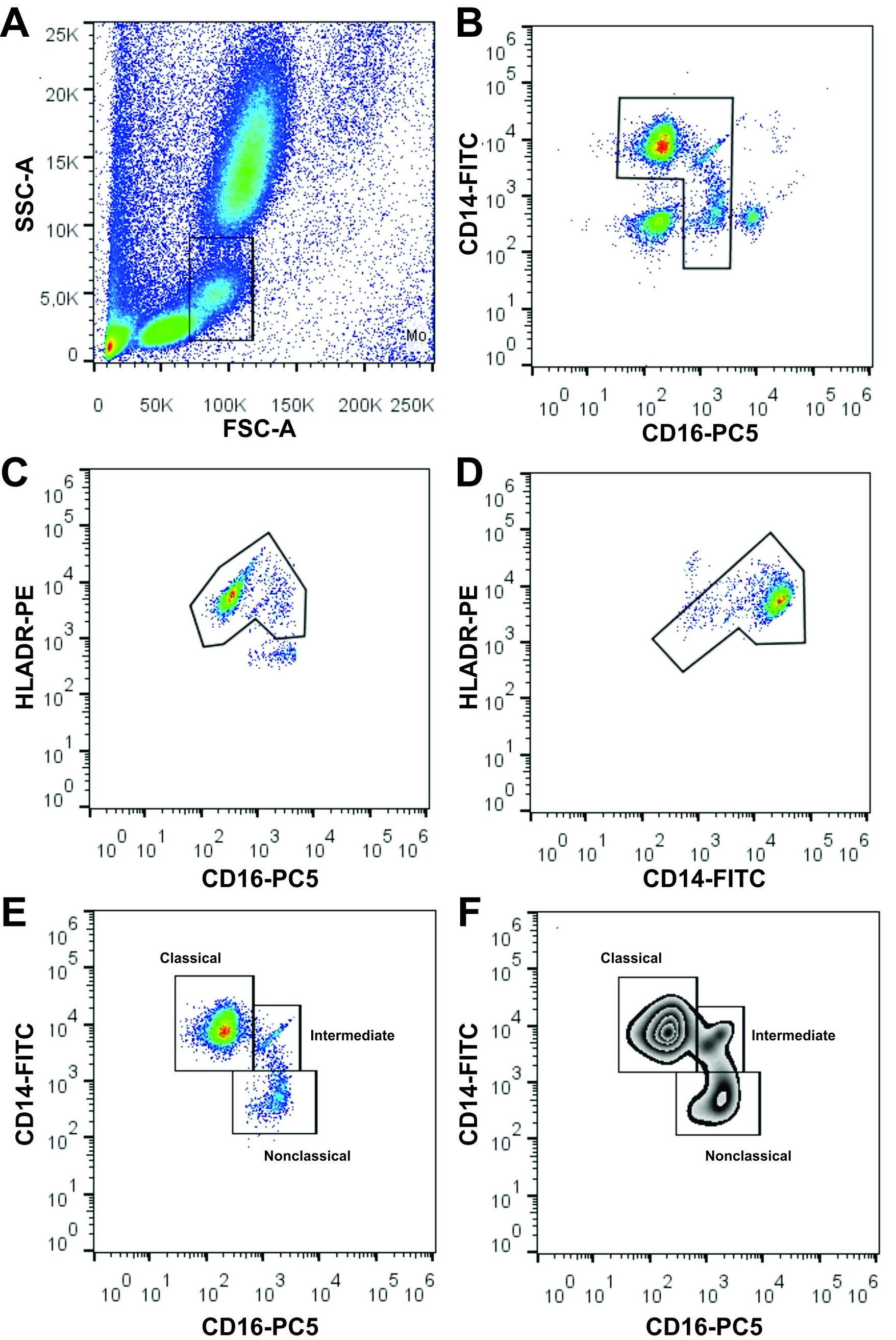

Supplement: S2 Fig — (A) FS vs. SS plot: Wide selection of monocytes depending on FS/SS properties. (B) Pseudocolor CD16 vs. CD14 plot: Gating to select monocytes depending on characteristic "inverted L" shape. (C) Pseudocolor CD16 vs. HLA-DR plot: Gating to select HLA-DR+ cells and to remove NK cells. (D) Pseudocolor CD14 vs. HLA-DR plot: Gating to exclude B cells (HLA-DRhigh/CD14low). (E) Pseudocolor CD16 vs. CD14 plot: Gating to select classical (CD14highCD16-), intermediates (CD14highCD16+) and non-classical (CD14lowCD16+) monocytes. (F) Zebra CD16 vs. CD14 plot: Selected monocytes redisplayed on CD16 vs. CD14 zebra plot to visualize monocyte subsets. (TIF) [file pone.0241097.s003.tif]
